# Dual‐Targeting Nanoliposome Improves Proinflammatory Immunomodulation of the Tumor Microenvironment

**DOI:** 10.1002/adhm.202302046

**Published:** 2023-09-21

**Authors:** Zili Gu, Candido G. da Silva, Sen Ma, Qi Liu, Timo Schomann, Ferry Ossendorp, Luis J. Cruz

**Affiliations:** ^1^ Department of Radiology Leiden University Medical Center Leiden 2333 ZA The Netherlands; ^2^ Department of Ophthalmology Leiden University Medical Center Leiden 2333 ZA The Netherlands; ^3^ Department of Internal Medicine University of Texas Southwestern Medical Center Dallas TX 75390 USA; ^4^ Department of Vascular Surgery Leiden University Medical Center Leiden 2333 ZA The Netherlands; ^5^ Department of Immunology Leiden University Medical Center Leiden 2333 ZA The Netherlands

**Keywords:** checkpoint blockade, immunotherapy, liposomes, nanomedicines, tyrosine kinase inhibitors

## Abstract

Immunotherapies targeting immune checkpoints have revolutionized cancer treatment by normalizing the immunosuppressive microenvironment of tumors and reducing adverse effects on the immune system. Indoleamine 2,3‐dioxygenase (IDO) inhibitors have garnered attention as a promising therapeutic agent for cancer. However, their application alone has shown limited clinical benefits. Cabozantinib, a multitarget tyrosine kinase inhibitor, holds immunomodulatory potential by promoting infiltration and activation of effector cells and inhibiting suppressive immune cells. Despite its potential, cabozantinib as a monotherapy has shown limited efficacy in terms of objective response rate. In this study, IDO‐IN‐7 and cabozantinib are coencapsulated into liposomes to enhance tumor accumulation and minimize adverse effects. The liposomal combination exhibits potent cytotoxicity and inhibits the function of IDO enzyme. Furthermore, the dual‐targeted treatment effectively inhibits tumor development and reverses the suppressive tumor microenvironment by regulating both adaptive and innate branch of immune system. This is evidenced by pronounced infiltration of T cells and B cells, a decrease of regulatory T lymphocytes, a shift to a proinflammatory phenotype of tumor‐associated macrophages, and increases levels of neutrophils. This is the first developed of a liposome‐delivered combination of IDO inhibitors and cabozantinib, and holds great potential for future clinical application as a promising anticancer strategy.

## Graphical abstract

A clinical standard liposome system is employed to simultaneously deliver cabozantinib and indoleamine 2,3‐dioxygenase inhibitor, and thereby exhibit improved anti‐tumor efficacy, which shows high biocompability and safety. After PEGlyation on the surface of the liposomes, high tumor accumalation can be achieved due to the prolonged ciculation, leading to boosted immune response and regulated tumor microenvironment.

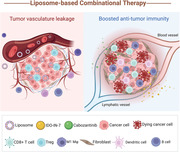

## 1 Introduction

Recently, the cancer treatment paradigm has evolved rapidly with the approval of several checkpoint inhibitors, which revealed that immunotherapy had have exhibited superior efficacy to chemotherapy alone.^[^ ^1^, ^2^ ^]^ Among the immune checkpoint blockers, an inhibitor of indoleamine 2,3‐dioxygenase has emerged as a promising therapeutic agent for cancer treatment. Indoleamine 2,3‐dioxygenase (IDO) is an immunoregulatory enzyme generated by tumor cells and myeloid‐derived suppressor cells in response to IFN‐γ signaling, catalyzing the oxidative metabolism of tryptophan to kynurenine.^[^ ^3^ ^]^ The depletion of tryptophan and accumulation of kynurenine in the tumor microenvironment results in suppression of effector T cells, while promoting the function of T regulatory cells and myeloid‐derived suppressor cells (MDSCs), leading to the formation of an immunosuppressive microenvironment.^[^ ^4^, ^5^, ^6^, ^7^ ^]^ Therefore, IDO inhibitors can neutralize the immunosuppressive tumor microenvironment and stimulate the host immune system.^[^ ^8^ ^]^ Although, several IDO inhibitors are currently undergoing clinical trials, there are limitations to its use as monotherapy. Generally, only a limited fraction of cancer patients achieves an objective response to checkpoint blockade, while many patients do not experience clinical benefits.^[^ ^9^, ^10^ ^]^ It has been reported in a study that no objective responses were reported for IDO inhibitor monotherapy among 52 patients with several tumor types.^[^ ^11^ ^]^ Recently, combinations of checkpoint inhibitors therapies have shown significant improvements in multiple cancer types compared with checkpoint inhibitor monotherapy,^[^ ^12^, ^13^, ^14^ ^]^ which encourages us to develop combinational strategies that broaden the use of checkpoint inhibitors for cancer therapy

The abnormal tumor vasculature creates a hypoxic microenvironment that polarizes inflammatory cells toward immune suppression. Combining antivascular endothelial growth factor (VEGF)/vascular endothelial growth factor receptor (VEGFR) agents with checkpoint blockade, emerged as a viable strategy to advance the immunotherapy paradigm.^[^ ^15^ ^]^ VEGF is a prime example of signaling pathways promoting cancer treatment escape. Therefore, targeting anti‐VEGF/VEGFR represents a key strategy for antiangiogenic therapy. Anti‐VEGF therapy also restores the interaction of endothelial‐T cells through the effects on vascular cell adhesion molecule 1 and intercellular adhesion molecule 1, leading to effective T cells infiltration into tumor tissue.^[^ ^16^ ^]^ Hence, antiangiogenic treatment benefits the trafficking of tumor‐specific T cells and other immune effectors.^[^ ^17^ ^]^ In addition to their impacts on tumor vasculature, VEGF inhibitors display immunomodulatory potential, including the ability to promote infiltration and activation of effector cells and to inhibit suppressive immune cells.^[^ ^18^ ^]^ Studies evaluating checkpoint inhibitors with VEGF/VEGFR‐targeted therapies have shown clinically meaningful activity.^[^ ^19^, ^20^, ^21^ ^]^

Cabozantinib, a small multitargeted tyrosine kinase inhibitor (TKI) with a VEGF inhibitory profile, has emerged as a potential partner for checkpoint inhibitor therapy. Cabozantinib also targets MET signaling, which is observed in response to anti‐VEGF therapy. The cMET pathway is a likely mechanism exploited by cancer cells to escape from antiangiogenic therapy.^[^ ^22^ ^]^ The hypoxic tumor microenvironment stimulates MET expression, which increases tumor invasion and metastasis after selective inhibition of VEGF signaling. The phenomenon could be reduced by concurrent inhibition of the VEGF and MET signaling pathways.^[^ ^23^, ^24^ ^]^ In addition, cabozantinib potently inhibits AXL, which suppresses major histogram complex (MHC) I expression and dampen antigen presenting cell (APC) function.^[^ ^25^ ^]^ A murine study has demonstrated that cabozantinib could render tumor cells more sensitive to immune‐mediated killing and regulate the antitumor immunity to generate a more permissive immune environment.^[^ ^26^ ^]^ However, in the clinic, cabozantinib monotherapy did exhibit improved progression‐free survival and overall survival, but did not have a notable objective response rate.^[^ ^27^, ^28^ ^]^ Due to the properties mentioned above, there is a solid rationale to combine cabozantinib and IDO inhibitors to improve cancer therapy.

**Figure 1:**
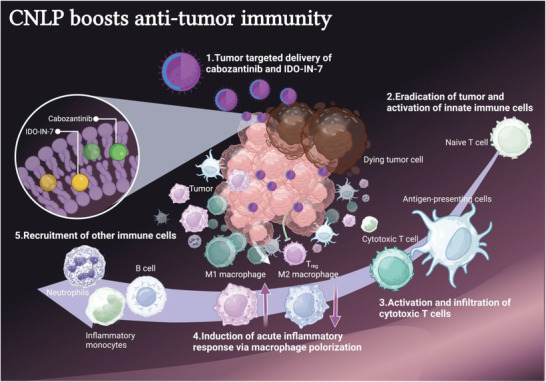
Schematic illustration of liposome‐mediated combinational therapy. The cabozantinib and IDO‐IN‐7 are enclosed in the lipid bilayer of liposomes. These liposomes can passively target tumor tissues after long circulation. Once entering tumor, on the one hand, they can kill tumor cells directly by interfering the metabolism of tumor cells. On the other hand, they can also generate a more permissive tumor microenvironment by activating innate immune responses and strengthening adaptive immune responses. In detail, CNLP treatment significantly recruited more effector CD8^+^ T cells, eliminated the number of T_reg_, enhanced the influx of neutrophils and inflammatory monocytes into the tumor, and induced a shift from the anti‐inflammatory M2 phenotype toward a proinflammatory M1 phenotype in the tumor microenvironment.

This study selected a triple‐negative breast cancer (TNBC) model to explore the synergetic effect between cabozantinib and IDO inhibitors, due to the high expression of both VEGF and IDO in TNBC.^[^ ^29^, ^30^ ^]^ Both cabozantinib and IDO inhibitors are small molecular drugs with poor solubility in water. Therefore, a clinical standard liposome system was employed to simultaneously deliver cabozantinib and IDO inhibitors, and thereby achieve improved efficacy, which showed high biocompatibility and safety. After PEGlyation on the surface of the liposomes, high tumor accumulation could be achieved due to the enhanced targeting ability, thereby leading to improved efficacy and decreased off‐target toxicity. The rationale of this study is illustrated in **Figure** **1**. In contrast to the corresponding monotherapies and combination therapy using conventional drug formulations, a single treatment with liposomes coloaded with the two drug results in prolonged local tumor control in a subcutaneous TNBC mouse model.

## 2 Results and Discussion

### 2.1 Liposome Design and Characterization

**Figure 2:**
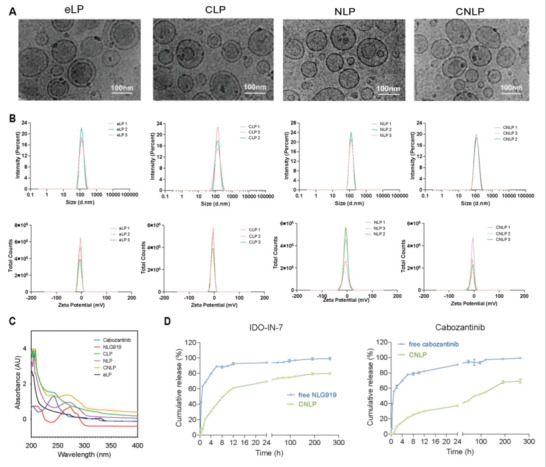
Physiochemical characterization of liposomes. A) Cyro‐EM photographs of different liposomes: empty liposomes (eLP); cabozantinib‐loaded liposomes (CLP). IDO‐IN‐7‐loaded liposomes (NLP); coloaded liposomes (CNLP). B) Size and zeta potential distribution of liposomes. C) UV‐absorbance of free drugs and liposomes. D) In vitro cumulative release behaviors of free drugs and liposomes.

The coloaded liposome (CNLP) was prepared with a thin‐film hydration method. Cabozantinib and IDO‐IN‐7 were encapsulated into the lipid bilayer since both of them are highly hydrophobic. In this design, liposomes act to protect the payload from degradation and clearance from the blood, decreasing hydrolysis and unsatisfied release before entering the target site. Once entering the tumor site, the lipophilic property of liposomes can sufficiently assist the uptake of payload and their subsequent function. Cyro‐EM displayed that all the liposomal formulations possessed a spherical appearance and uniform dispersion (**Figure** **2**A), which was further confirmed by transmission electron microscopy (TEM) under multiple magnifications (Figure S1A, Supporting Information). These photos also showed that the majority of liposomes were large unilamellar vesicles after extrusion. As Table S1 (Supporting Information); and Figure 2B show, the diameter of empty liposomes (eLP), cabozantinib‐loaded liposomes (CLP); IDO‐IN‐7‐loaded liposomes (NLP), and CNLP were 124.43 ± 2.27, 122.97 ± 1.56, 124.07 ± 0.69, and 115.53 ± 2.35 nm, respectively, with a low poly density index (PDI) of 0.07 ± 0.02, 0.07 ± 0.03, 0.05 ± 0.02, and 0.14 ± 0.01, indicating proper size for passive targeting and high dispersity of liposomes. Upon encapsulation, drug‐loaded liposomes exhibited a similar ultraviolet absorbance to that of the free drugs, suggesting rare damage to the drug structure (Figure 2C). The EE% of cabozantinib and IDO‐IN‐7 was ≈80% and ≈90%, respectively, and DL% was determined to be ≈4% (wt%) by UPLC (Table S2, Supporting Information). To improve stability and enhance their circulation time in the blood, all the liposomes were PEGylated. Consequently, their charge was near neutral (Table S1, Supporting Information), which would protect the liposomes from clearance by the reticuloendothelial system (RES) and enhance their therapeutic effects. Our data of the stability assay showed that these liposomes exhibited stable structures in phosphate‐buffered saline (PBS), 10% and relatively stable in after 1 h in 50% serum (Figure S1B, Supporting Information), facilitating sustained release of the loaded drug in vivo.

Next, we investigated the kinetic behavior of liposomes in physiological environment (pH 7.4) at 37 °C. Notably, CNLP revealed a distinctly sustained release profile with different kinetics for both drugs (Figure 2D). The free drugs exhibited a burst release within 1 h of incubation and reached to a plateau (>80%) within 6 h. In contrast, cabozantinib and IDO‐IN‐7 were released from liposomes over a period of several days, which was much slower than the free drugs, especially cabozantinib. Upon release, cabozantinib is liberated to initiate inhibition of multiple kinases, reprogram the tumor microenvironment, increase infiltrating lymphocytes, and synergize with immunotherapy to control tumor growth, which provides an opportunity for TNBC treatments.^[^ ^31^, ^32^ ^]^ In addition, the release of IDO‐IN‐7 into tumor site, may also beneficially complement the cytotoxicity of cabozantinib by the induction of mammalian target of rapamycin complex (mTOR) proliferation signals.^[^ ^33^ ^]^ An enhanced mTOR activity reactivated by IDO inhibitors may boost the efficacy of cytotoxic therapies, reactivate T‐cell to overcome the tumor immune escape, and beneficially complement an anticancer therapy.^[^ ^34^, ^35^ ^]^

Therefore, together with the long circulation ability, these sustainable liposomes could shield cargo against clearance and maximum drug accumulation inside the tumor. Our analysis indicated that CNLP is well designed and prepared for efficient drug delivery, which is beneficial for intracellular drug accumulation in vitro and in vivo.

### 2.2 Inhibitory Capacity of Liposomal Formulations

**Figure 3:**
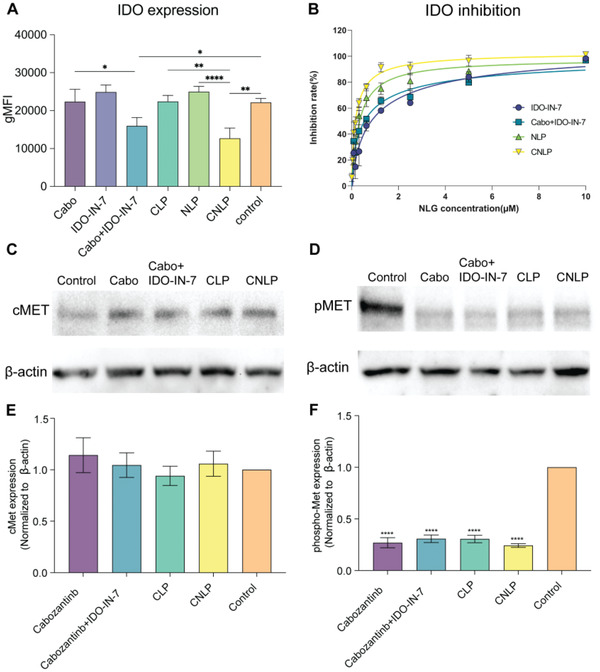
Assessment of inhibitory potency of liposome formulations. A) Quantification of IDO expression on 4T1 cells after incubation with different treatments for 24 h. B) The inhibition rate of IDO enzyme activity after incubation with free drugs and liposomes for 48 h. C) cMET expression after treated with free drugs and liposomes, measured by western blot. D) pMET expression after treated with free drugs and liposomes, measured by western blot. E) Normalized cMET expression from western blot. F) Normalized pMET expression from western blot (Significant difference is compared between control and other groups). (*n* = 3, results were shown in mean ± S.D., *, *p* < 0.05, **, *p* < 0.01, ***, *p* < 0.001, ****, *p* < 0.0001).

First, we investigated the IDO expression on 4T1 breast cancer cells after treatment. As shown in **Figure** **3**A, after treatment with monotherapy, either free drugs or mono drug‐loaded liposomes (CLP and NLP), exhibited nearly no change, with similarity to the control group. No change in the expression is expected, since IDO‐IN‐7 inhibits the function of IDO, not its expression. Strikingly, we found that the combination of cabozantinib and IDO‐IN‐7 slightly downregulated the expression of the IDO enzyme. Although the difference was not significant and the mechanism was unclear, this effect was still enhanced in the dual drug‐loaded CNLP group. This finding indicates that the combinational strategy can mildly decrease the expression of IDO enzyme as well as to restrain its function, supporting our hypothesis to combine these two drugs for improved anticancer effects.

Subsequently, to ensure the bioactivity of drugs after loaded into liposomes, we also examined the inhibition of IDO's enzymatic activity in vitro. Since IDO‐mediated depletion of tryptophan and generation of kynurenine result in inhibition of T cell activity as well as induction of T cell apoptosis,^[^ ^36^ ^]^ the overexpression of IDO in tumor cells is connected to the reduced overall survival of multiple cancer types. To this end, the tumor cells were pretreated with IFN‐γ to ensure high IDO expression to mimic the environment from the tumor.^[^ ^36^, ^37^, ^38^ ^]^ As shown in Figure 3B, free IDO‐IN‐7 as well as combination of the free drugs inhibited the IDO activity in a dose‐dependent manner with an IC50 of 0.83 and 0.53 µm. However, NLP and CNLP exhibited IC50 of 0.25 and 0.18 µm. Both NLP and CNLP exhibited higher capability of inhibiting the conversion of tryptophan to kynurenine than free IDO‐IN‐7 and the combination of free drugs. This stronger inhibition may be attributed to the higher level of IDO‐IN‐7 induced by efficient uptake of liposomes inside the tumor cells where IDO enzyme locates. We also sought to determine the bioactivity of cabozantinib, using Western blot analysis we determined that cells treated with CLP and CNLP exhibited unaffected expression of cMET (Figure 3C,E) and significantly reduced levels of phosphorylation of the protein cMET (pMET) (Figure 3D,F) compared to control, indicating a decrease in this pathway related downstream to tumor invasion and metastasis. In the clinic, high levels of pMET were commonly seen in many breast cancer subtypes and correlated with poor prognosis. Notably, the more pronounced inhibition of phosphorylation in cells treated with CNLP suggests a stronger inhibitory effect of liposomal formulation on tumor progression.

### 2.3 CNLP Enhances Antitumor Effects In Vitro and In Vivo

**Figure 4:**
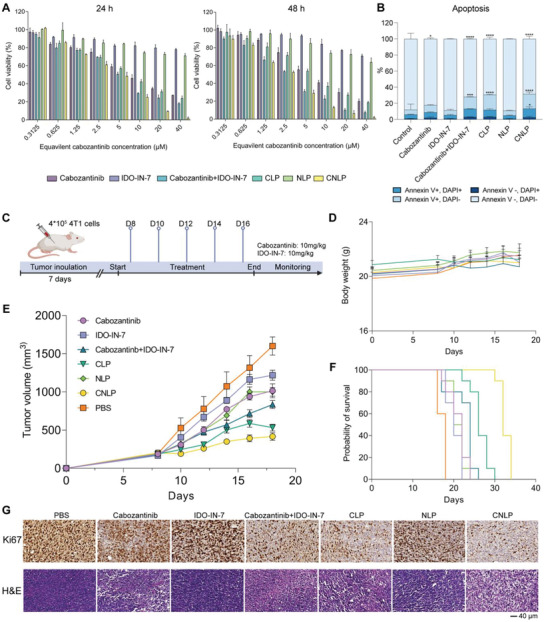
Antitumor effect in the 4T1 breast cancer model treated with different formulations in vitro and in vivo. A) Cytotoxicity assay of 4T1 cells treated with cabozantinib, IDO‐IN‐7, free combination, CLP, NLP, CNLP with various dose and times for 24 and 48 h (*n* = 3). B) Apoptosis analysis of 4T1 cells induced by PBS, eLPs, cabozantinib, IDO‐IN‐7, free combination, CLP, NLP, and CNLP (*n* = 3, results were shown in mean ± S.D., *, *p* < 0.05, **, *p* < 0.01, ***, *p* < 0.001, ****, *p* < 0.0001). C) Schematic illustration of therapeutic schedule against 4T1 tumors. D) Average changes of mouse body weight after multiple treatments (*n* = 8). E) The tumor growth curve of 4T1 tumor‐bearing mice after multiple treatments (*n* = 8). F) Survival rates of 4T1 tumor‐bearing mice after multiple treatments (*n* = 8). G) Histological Ki67 & H&E staining of tumor material collected at day 17 after tumor inoculation. (Scale bar = 40 µm).

After assessment of the quality of the liposomes, the formulations were carefully examined for safety and efficiency both in vitro and in vivo. To ensure good biocompatiblility of the formulations, we first tested the cytotoxicity of eLP on 4T1 cells by means of a MTS assay. The results showed a survival rate of over 90% even at 200 µg mL^−1^ lipid concentration (Figure S2A, Supporting Information), indicating excellent biocompatibility of liposomes for further studies. As for drug‐treated cells, all the groups containing cabozantinib inhibited the proliferation of tumor cells in a dose‐ and time‐dependent manner, while IDO‐IN‐7 monotherapy was nontoxic (**Figure** **4**A). In detail, free cabozantinib and the free combination both induced moderate proliferation inhibition of 4T1 cells with IC_50_ of 8.5 and 5.0 µm after 24 h of incubation, compared to the control group. The higher cytotoxicity of the free compound combination may be attributed to the proliferation signals induced by activation of mTOR via IDO‐IN‐7.^[^ ^39^ ^]^ The results of our study showed that both CLP and CNLP exhibited a higher rate of proliferation inhibition compared to the corresponding free drugs. Specifically, the IC_50_ values for CLP and CNLP were 6.9 and 3.7 µm, respectively, after 24 h of incubation. This enhancement in antiproliferation effect could be attributed to the increased internalization of the drugs within tumor cells, resulting from the liposomal encapsulation. Furthermore, since cabozantinib has been reported to disrupt cell growth via inducing apoptosis, we also investigated the cell apoptosis after treatment (Figure 4B; and Figure S2B, Supporting Information). In consistence with the MTS results, the cabozantinib‐treated groups induced moderate cell apoptosis, whereas the control eLP and IDO‐IN‐7‐treated groups showed almost no cytotoxicity. Among groups with dying cells, liposome groups induced higher apoptosis especially early apoptosis (Annexin V^+^ DAPI^−^), which indicates excellent drug delivery efficiency of the liposomes. Taken together, these data reflect the efficient cytotoxic effect of combinational liposomes in vitro.

Next, we established a subcutaneous 4T1 TNBC animal model in BABL/c mice to investigate the suppression of tumor development upon liposomal treatment. At day 8 after tumor cells inoculation, the mice were randomly divided, followed by i.v. injections as indicated in Figure 4C. As shown in Figure 4D, the average body weight of mice with the free combination was decreased after continuous administration. However, mice treated with CNLP displayed a stably increase in body weight, suggesting that the liposomes can reduce systemic toxicity of cytotoxic drugs. After the treatment with IDO‐IN‐7, there were slightly higher anti‐tumor effects in comparison to the control, while elevated but still limited, suppression of tumor growth was found in the NLP group (Figure 4E). Cabozantinib showed a moderate effect (0.63‐fold tumor growth of control) in inhibiting the growth of 4T1 tumors, which was comparable to that of NLP. However, the synergistic effect was found in the combination groups, both for the free drugs (0.51‐fold tumor growth of control) as well as liposome‐encapsulated drugs (0.22‐fold tumor growth of control), which indicated the beneficial effect of the combinational strategy. Notably, CLP, as a monotherapy, gained more effective antitumor effects in comparison to free cabozantinib, probably due to the higher drug accumulation induced by liposomes. Furthermore, the CNLP group exhibited the best antitumor effects among all the groups without weight loss. Associated with the efficient tumor inhibition, the survival benefit of liposomal treatment was also observed in 4T1 tumor‐bearing mice, especially in CNLP groups. This significantly prolonged the survival time of tumor‐bearing mice which indicates that the liposome‐mediated IDO and VEGF/MET/AXL inhibition can inhibit tumor progression better than monotherapy/free combination.

In addition, we performed immunohistochemical staining to further investigate the comprehensive antitumor effect of liposomes in vivo. Based on Ki67 and H&E staining, the highest degree of cell death and lowest cell proliferation were observed in the CNLP group, which was reflected by chromatin condensation & cell/tissues structure shrinkage in H&E observation and downregulated Ki67 expression (Figure 4G). This suggests better alleviation of the tumor burden induced by CNLP. The effective antitumor growth effects of the liposome‐based combinational therapy might be explained as follows: First, the sustained release and passive targeting ability of liposomes effectively improved the tumor penetration, consequently improving the bioavailability of payloads. Second, the utilization of liposome‐based combination largely improves the unsatisfied therapeutic outcome of chemotherapy including the limited tumor accumulation, side effects, as well as insufficient immune evasion. Also the low response rate of effective immunotherapy which includes the insufficient exposure of tumor associated antigens and IDO‐mediated resistance. Except for sensitizing tumor cells to chemotherapy and reducing the immune resistance by reducing IDO inhibition, this strategy may also effectively activate antitumor immune responses in the tumor microenvironment assay. In summary, our liposome‐based delivery of IDO‐IN‐7 and cabozantinib showed to be an effective combination and delivery system to optimally inhibit tumor growth in a murine model of 4T1 breast cancer.

### 2.4 Liposomes Improve Cellular Internalization and Biodistribution

**Figure 5:**
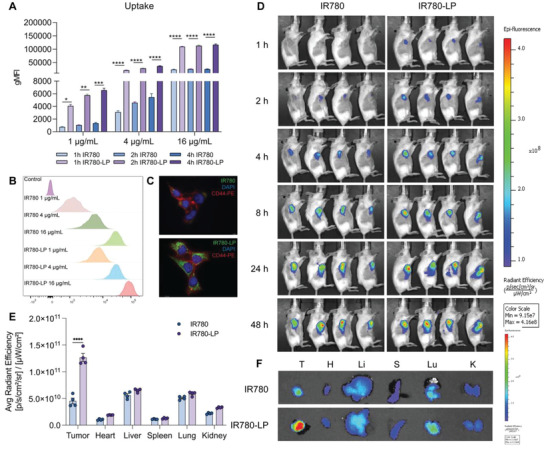
Tumor targeting ability of liposomes. A) Quantification of intracellular uptake after incubated with free IR780 and IR780‐LP in 4T1 cells in vitro (*n* = 3). B) Histogram of intracellular uptake of free IR780 and IR780‐LP by 4T1 cells after 4 h after incubation in vitro. C) Fluorescent images of cells treated with free IR780 and IR780‐LP 4 h after i.v. incubation. D) IVIS images of free IR780 and IR780‐LP distribution in 4T1 tumor‐bearing mice. E) Normalized fluorescence intensity of the tumor and major organs (*n* = 4). F) IVIS images of free IR780 and IR780‐LPs in hearts (H), livers (Li), spleens (S), lungs (Lu), kidneys (K), and tumors (T) at 48 h postinjection (*n* = 4, results were shown in mean ± S.D., *, *p* < 0.05, **, *p* < 0.01, ***, *p* < 0.001, ****, *p* < 0.0001).

The strong antitumor effects lead us to explore the liposome‐mediated cellular uptake and tumor targeting ability. For quantification and real‐time monitoring in the uptake and biodistribution assays, liposomes were labeled with a fluorescent dye (IR780‐LP). First, we examined the cellular internalization of liposomes in vitro by means of flow cytometry. As shown in **Figure** **5**A, both free IR780 and IR780‐LP exhibited dose‐ and time‐dependent internalization. Compared to free IR780, IR780‐LP was taken up by tumor cells with a significantly higher efficiency (Figure 5B), especially at low concentration (Figure S3, Supporting Information). Fluorescence microscopy demonstrated higher accumulation of IR780 (green) located in the cytoplasm than free IR780 (Figure 5C), implying a better drug delivery efficiency of our liposomes. Taken together, our data indicates that liposomes provide an effective platform to deliver hydrophobic drugs into the target site with high efficiency.

Since the liposome‐mediated drug accumulation in the tumor site plays a fundamental role in the treatment efficiency in vivo, we noninvasively tracked the drug distribution in real‐time via the IVIS Spectrum In Vivo Imaging System. When the tumor volume reached 200 mm^3^, free IR780 or IR780‐LP were intravenously injected into mice at an identical dose of 100 µg kg^−1^. In vivo fluorescence images demonstrated both IR780 and IR780‐LP gradually accumulated in the tumor tissue, but the fluorescence intensity of the liposome‐treated group was much stronger than that of free drug group (Figure 5D). Within the first 2 h, the signal of the free IR780 group was negligible, whereas a clear signal was detected in the IR780‐LP group. Moreover, the accumulation of IR780 was still strong even after 48 h, which is in line with the long circulation ability and sustained release profile of liposomes. Furthermore, the ex vivo distribution analysis in the tumor and other major organs showed that IR780‐LP displayed a 2.8‐fold higher intertumoral accumulation than free IR780 (Figure 5E,F), verifying the improved drug accumulation and retention within the tumor due to the (passive) targeting ability of liposomes. These data indicate that liposomes can passively target tumor tissue and exert high drug accumulation, which lays a solid foundation for their effective use for in vivo treatment.

### 2.5 CNLP Treatment Regulates the Levels of Circulating CD3^+^, CD4^+^, CD8^+^ T Cells, B Cells, and Cytokines

The presence of solid tumors can influence circulating lymphocytes, which has been shown for multiple cancer types, such as colorectal and breast cancer.^[^ ^40^ ^]^ It was reported that maintaining the number of circulating inflammatory cells can enhance the response to chemotherapy and that a lymphocyte‐mediated immune response may play a positive role in the eradication of tumor cells.^[^ ^41^ ^]^ After specific therapy, tumor cells release cues such as DAMPs, which are presented to naive T cells by antigen‐presenting cells (APC) for priming and activation. Subsequently, activated lymphocytes leave the lymph node, enter the tumor tissue via the blood circulation, and exert antitumor activities.

**Figure 6:**
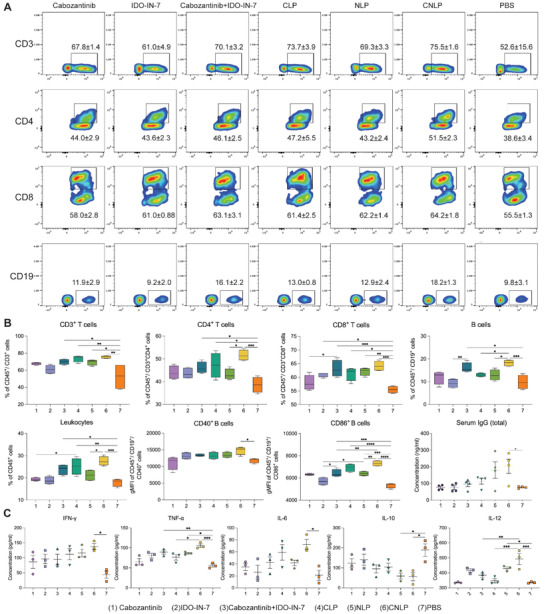
Blood analysis of 4T1 tumor‐bearing mice after treatments. A) Circulating total numbers of CD3^+^, CD3^+^CD4^+^, and CD3^+^CD8^+^ T cells and CD19^+^ B cells in the blood. B) The ratio of lymphocytes and serum IgG in the blood after treatments (*n* = 4). C) The levels of cytokines in the peripheral blood after the administration of different treatment (*n* = 3, results were shown in mean ± S.D., *, *p* < 0.05, **, *p* < 0.01, ***, *p* < 0.001, ****, *p* < 0.0001).

Given the long circulation ability and high specificity of liposomes, we next measured the immune response in the blood circulation at day 16 in 4T1 breast‐tumor‐bearing mice after receiving different treatments. The Gating strategies was shown in Figures S4 and S5 (Supporting Information). As displayed in **Figure** **6**A,B, the CNLP group exhibited significantly elevated levels of circulating CD3+, CD4+, as well as CD8+ T cells compared to the control group. In addition, the CNLP group showed stronger recruitment of T lymphocytes compared to the free combination, although it was not significant. Notably, the population of B cells of CNLP‐treated animals was significantly higher (≈2‐fold) than in the control group, especially in CD86^+^ B cells (Figure 6B), which is consistent with the elevation of IgG release. This indicates that the co‐encapsulation of IDO‐IN‐7 and cabozantinib could strengthen the recruitment of lymphocytes from lymph nodes into the tumor tissue. Additionally, immune cytokines, such as IFN‐γ, TNF‐α, IL‐6, IL‐10, and IL‐12, also have a strong connection to the suppressive tumor microenvironment. As shown in Figure 6C, the levels of IFN‐γ, TNF‐α, IL‐6, and IL‐12 in the liposome‐treated group were higher than with free drugs, while the level of IL‐10 in liposome groups was lower than with free drugs, either in monotherapy or combination. Among the liposomal treatments, CNLP exhibited higher activity regarding the increase of stimulatory cytokines and elimination of suppressive cytokines. These results together indicates that CNLPs could boost the antitumor immunity and reshape the tumor microenvironment by recruiting the immune cells and regulating the level of cytokines, which further reinforce the efficacy of the combinational therapy against breast cancer.

### 2.6 CNLP Boosts Lymphocyte Influx and Function in the Tumor Microenvironment, Spleen, and Lymph Node

**Figure 7:**
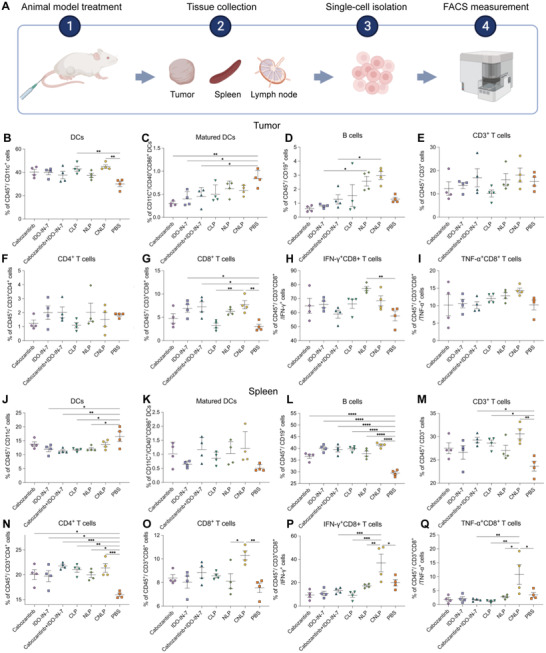
The level of lymphocyte populations in the tumor microenvironment and spleen after treatments of 4T1 tumor‐bearing mice. A) Illustration of sample preparation and measurements. A–I) Percentage of DCs B), matured DCs C), B cells D), CD3^+^ T cells E), CD4^+^ T cells F), CD8^+^ T cells G), IFN‐γ^+^CD8^+^ T cells H), TNF‐α^+^CD8^+^ T cells I) in the tumor microenvironment. J–Q) Percentage of DCs J), matured DCs K), B cells L), CD3^+^ T cells M), CD4^+^ T cells N), CD8^+^ T cells O), IFN‐γ^+^CD8^+^ T cells P), TNF‐α^+^CD8^+^ T cells Q) in the spleen (*n* = 4, results were shown in mean ± S.D., *, *p* < 0.05, **, *p* < 0.01, ***, *p* < 0.001, ****, *p* < 0.0001).

To elucidate the mechanisms underlying the potent tumor inhibition effects of these liposome‐mediated combinational therapy, we investigated the infiltration of lymphatic cells and their function in the tumor microenvironment, spleen, tumor‐draining lymph node (dLN), and nontumor draining lymph nodes (ndLN) after a completed treatment with CNLP and analyzed by means of flow cytometry (**Figure** **7**A). In previous coculture assay (DCs + tumor cells), we found that CNLP‐treated tumor cells could potently enhance the activation of DC maturation (Figure S6, Supporting Information). Giving this encouraging data, we analyzed the frequency of DC maturation in vivo after treatment since it is the prerequisite for an effective T cell activation. The CNLP group exhibited 2.4‐ and 2.8‐fold higher level of matured DCs (CD11c^+^CD40^+^CD86^+^) than the corresponding control group in the dLN and ndLN, respectively (Figure S7A,B, Supporting Information). This finding shows that CNLPs can significantly promote DC maturation in the lymph nodes. It is worth noting that, although the level of CNLP‐induced DC maturation within the tumor microenvironment was lower than the control group, it was still higher than all the other groups (Figure 7B,C). Similar results were observed in the spleen (Figure 7J,K). This indicates that the occurrence of immune resistance against the free combination could be attributed to the liposomal delivery to some degree. Besides, B cells are also an important type of APCs, their infiltration was also examined in this study. As shown in Figure 7D; and Figure S7 (Supporting Information) CNLPs induced significantly higher B cell infiltration into the tumor microenvironment and all the investigated lymphatic organs. Together with the higher maturation of DCs, these APCs could perhaps provide enough signal for T cells to enhance further cancer cell killing ability.

Next, we investigated the APC‐mediated immune response by measuring the intratumoral infiltration of T lymphocytes as well as their engagement in the spleen and lymph nodes. First, flow cytometric detection revealed that CNLP elicited more leukocytes (CD45^+^) and total T lymphocytes (CD45^+^CD3^+^) than the control group (Figure S8 (Supporting Information); and Figure 7E,M). In particularly, CNLP promoted a 2.6‐fold higher intratumoral infiltration of CD8^+^ T cells than the control group (1.4‐, 1.4‐, and 1.1‐fold higher to that of spleen, dLN, and ndLN, respectively), while enhancing the amount of CD4^+^ T cells in all the lymphatic organs (Figure 7F,G,N,O; and Figure S8, Supporting Information). This increase of lymphocytes demonstrated the effective acute and memory immune response that had been facilitated by CNLP. Furthermore, to understand the mechanism of antitumor immunity in more detail, we also evaluated the function of cytotoxic T lymphocytes (CTLs) by examining the level of Granzyme B^+^, IFN‐γ^+^, and TNF‐α^+^ CD8^+^ T lymphocytes. Our data revealed that the control group had 57.6%, 19.9%, and 4.5% IFN‐γ^+^ CD8^+^ T lymphocytes in the tumor microenvironment, spleen, and dLNs, respectively, whereas CNLP enhanced it to 68.5%, 37.0%, and 10.7%, respectively (Figure 7H,P; and Figure S7A, Supporting Information). In addition, CNLP treatment promoted the intratumoral infiltration of Granzyme B^+^ CD8^+^ T lymphocytes from 10.0% to 19.0% (Figure S9, Supporting Information). Besides, as for TNF‐α^+^ CD8^+^ T lymphocytes, CNLP also exhibited strong elevation, which raised a 1.4‐, 3.1‐, 4.2‐, and 1.3‐fold infiltration into the tumor microenvironment, spleen, dLNs, and ndLN (Figure 7I,Q). Similar outcomes were seen by the examination of cytokines extracted from the tumor tissues and analyzed via ELISA‐assays. The expression of inflammatory cytokines including IFN‐γ, TNF‐α, IL‐6, and IL12 were significantly upregulated by CNLP treatment and the IL‐10 suppressive cytokine was clearly downregulated (Figure S10, Supporting Information). Additionally, it has been reported that IDO inhibition can decrease the activity of regulatory T cells (T_reg_).^[^ ^42^ ^]^ Therefore, we further measured the population of T_reg_ in the tumor microenvironment and spleen after liposomal treatment. Upon monotherapy with CLP or NLP, the frequency of T_reg_ in the tumor microenvironment was 19.1% and 16.6%, respectively, which was much lower than the 35% of the control group (Figure S11, Supporting Information). However, after simultaneously delivery of cabozantinib and IDO‐IN‐7 by means of CNLP, the population of T_reg_ was strongly decreased to 7%, suggesting additional aid of CNLP in strengthening antitumor immunity via the elimination of T_reg_. Hence, the liposome‐mediated combinational therapy effectively boosted antitumor immunity and subsequently suppressed tumor development by raising the intratumoral infiltration as well as enhancing the activation of effector CTLs.

### 2.7 CNLP Treatment Modulates Antitumor Immunity and Induced an Inflammatory State in 4T1 Breast Tumor

**Figure 8:**
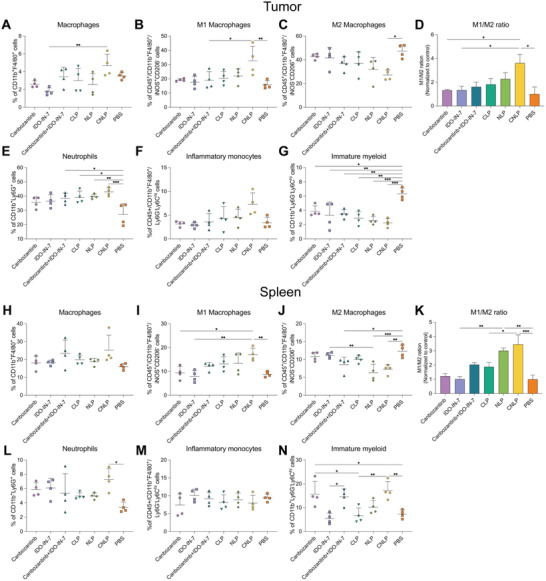
The level of myeloid populations in tumor and spleen after treatments. A–G) Percentage of macrophages A), M1 macrophages B), M2 macrophages C), M1/M2 ratio D), neutrophils E), inflammatory monocytes F), and immature myeloid G) in the tumor microenvironment. H–N) Percentage of macrophages H), M1 macrophages I), M2 macrophages J), M1/M2 ratio K), neutrophils L), inflammatory monocytes M), and immature myeloid N) in the spleen (*n* = 4, results were shown in mean ± S.D., *, *p* < 0.05, **, *p* < 0.01, ***, *p* < 0.001, ****, *p* < 0.0001).

Having demonstrated that the liposome‐based combinational therapy reduced the tumor burden of mice in vivo, and boosted T cell recruitment and activation, we further examined the myeloid immune response elicited by this treatment in the tumor microenvironment and related lymphatic organs. To this end, we inoculated mice with 4T1 cancer cells and treated them as described above (Figure 4C), then sacrificed the mice the day after the last liposome administration and collected tissues for analysis by means of flow cytometry. The gating strategy for myeloid population was showed in Figure S12 (Supporting Information). Within the myeloid population in the tumor, CNLP significantly increased the level of macrophages (CD11b^+^F4/80^+^), and exhibited a shift from an anti‐inflammatory M2 (CD11b^+^F4/80^+^iNOS^−^CD206^+^) to a more proinflammatory M1 (CD11b^+^F4/80^+^iNOS^+^CD206^−^) macrophage phenotype (**Figure** **8**A–D). Similar results were observed in the spleen and dLN (Figure 8H–K; and Figure S13A, Supporting Information), which appears also to be in line with the decrease of IL‐10 secretion (Figure S10, Supporting Information). As we know, proinflammatory M1 type macrophages are “educated” and differentiated toward M2 type macrophages upon interaction with a tumor, helping to establish an anti‐inflammatory milieu that inhibits the host's antitumor immune response and ultimately can aim in tumor immune evasion.^[^ ^43^, ^44^ ^]^ The polarization from M2 toward M1 phenotype within the tumor (Figure 8D) that can be mediated by our treatment liposomes demonstrated that CNLP could amplifying the infiltration of macrophages into tumor tissues and generate a more proinflammatory environment by increasing the number of M1 type macrophages. Besides, it has been reported that M2 type macrophages produce VEGF to stimulate angiogenesis and promote tumor cell survival and metastasis,^[^ ^45^, ^46^ ^]^ which might help to explain the potent antitumor effects of CNLP by inhibiting the level of M2 macrophages as well as their function.

Moreover, liposomal treatment significantly increased the level of neutrophils (CD11b^+^Ly6G^+^) compared to the free drug in the tumor microenvironment (Figure 8E). Among the liposome groups, CNLP showed the highest induction, increasing neutrophils to 43% compared to 27% in the control group. In the spleen and ndLNs, only CNLP exhibited significant difference in comparison to the control group, while no differences were observed in dLN (Figure 8L; and Figure S13, Supporting Information). In this study, all liposomes‐involving groups recruited higher level of inflammatory monocytes (CD11b^+^F4/80^+^/Ly6G^−^Ly6C^hi^) than the corresponding free drugs in the tumor microenvironment, dLN, and ndLN (Figure 8F; and Figure S13, Supporting Information), but these differences were not significant. Similar to neutrophils, CNLP was the most efficient to induce an acute inflammatory response, which supported tumor growth inhibition. Although the combination of cabozantinib and IDO‐IN‐7 augment the population of immature myeloid (CD11b^+^Ly6G^−^Ly6C^hi^) in the spleen (Figure 8N), the CNLP group could still significantly reduce the level of immature myeloid in the tumor, dLN, and ndLN (Figure 8G; and Figure S13, Supporting Information). We observed that liposomal monotherapy elicits moderate antitumor effects and regulates the antitumor immunity, however, these effects are still insufficient for the elimination of (all) suppressive immune cells and the activation of exhausted T cells to overcome immune evasion and resistance. This could be due to tumor cells overcoming acute inflammatory cytokines triggered by cabozantinib, or the depletion of kynurenine via IDO blockade was still insufficient even when IDO‐IN‐7 when was encapsulated into liposomes. Hence, it seems crucial to employ the combination of cabozantinib and IDO‐IN‐7 using liposomes for effective tumor growth inhibition. Taken together, our results revealed that CNLP orchestrated both lymphoid and myeloid populations in the tumor and lymphatic organs, suggesting the activation of innate immune responses and a potentiated adaptive immune response.

## 3 Conclusion 

In summary, in this study we coloaded tyrosine kinase inhibitor cabozantinib and IDO inhibitor IDO‐IN‐7 into liposomes (CNLP), which exhibited a round and uniform appearance, high stability, and sustained release ability. We observed the suppression of IDO expression as well as its activity, and the inhibition of phosphorylated cMET upon effective liposome‐based combinational therapy. By means of an in vitro cellular assay, we found that CNLP could efficiently inhibit tumor cell proliferation and effectively induce cancer cell apoptosis. It was further observed that CNLP exhibited the strongest antitumor effects in vivo and significantly prolonged the survival time of mice. We also demonstrated that CNLP possessed a passive targeting ability and penetrated into tumor tissues with high efficiency. Once at the target site, CNLP induced cell death and elicited strong DC maturation, which was shown both in vitro and in vivo. Upon tumor inoculation, our CNLPs displayed a comparatively better antitumor efficacy in the 4T1 triple negative breast cancer model. By exploring the mechanism behind the potent antitumor efficacy, we found that CNLPs created a relatively permissive tumor microenvironment by activating innate immune responses and strengthening adaptive immune responses. In more detail, CNLP treatment significantly recruited more infiltrating effector CD8^+^ T cells, depleted the level of T_reg_, enhanced the influx of neutrophils and macrophages into the tumor, and induced a shift from the anti‐inflammatory M2 phenotype toward a proinflammatory M1 phenotype in the tumor microenvironment. Given the above results, we have demonstrated that CNLP treatment enabled the suppression of tumor progression and boosted the immune response, which presents a promising and potential strategy for future triple negative breast cancer therapy.

## 4 Experimental Section

### Materials

Methoxy‐polyethylene glycol (MW 2000)‐distearoylphosphatidyl‐ethanoloamine (mPEG2000‐DSPE), 1,2‐dioleoyl‐sn‐glycero‐3‐phosphocholine (DOPC), and cholesterol were purchased from Avanti Polar Lipids (Alabaster, USA). Cabozantinib and IDO‐IN‐7 were purchased from MedChemExpress (NJ, USA).

### Cell Lines

The 4T1 cell line (mouse breast cells) was obtained by ATCC and cultured in RPMI1640 medium, supplemented 10% fetal bovine serum, 100 U mL^−1^ penicillin and 100 µg mL^−1^ streptomycin. The cells were incubated at 37 °C in the presence of 5% CO_2_.

### Animals

Female BALB/c mice were purchased from ENVIGO (Limburg, the Netherlands), and housed in pathogen‐free animal facilities at Leiden University Medical Center (LUMC). All experimental animals were 6–8 weeks old unless otherwise stated. The Dutch Animal Ethics Committee's Code of Conduct was followed in the design of the animal experiments and were carried out in accordance with Dutch animal welfare laws (project number: AVD116008045).

### Preparation

To prepare basic liposomes, DOPC: cholesterol: cabozantinib: IDO‐IN‐7: DSPE‐PEG2000 in a molar ratio of 7:3:1:1:0.5 were dissolved in chloroform and added to a glass flask. The chloroform solvent was evaporated at 35 °C to obtain a thin lipid film. The product was then dried overnight in a vacuum desiccator to completely remove residual chloroform from the lipid film. The dried film was hydrated with PBS (pH 7.4, 10 mm Na_2_HPO_4_, 1.76 mm KH_2_PO_4_, 137 mm NaCl, 2.7 mm KCl) at 40 °C for 30 min to yield the final lipid. The suspension was then forced through two stacked (400 + 100 nm) polycarbonate membrane (Whatman, NucleporeTM, GE healthcare, Little Chalfont, UK) using an Extruder (LIPEX Extruder, Northern Lipids Inc., Canada). The coloaded liposomes were stored at 4 °C until use. The IR780‐loaded liposomes (IR780‐LP) were prepared using the same method under dark conditions without cabozantinib or IDO‐IN‐7.

### Characterization

The particle size, size distributions, and ζ‐potentials of cabozantinib‐loaded liposome (CLP, IDO‐IN‐7‐loaded liposome (NLP) and coloaded liposome (CNLP) were measured on a Zetasizer Nano ZS90 instrument (Malvern Instruments, Malvern, London, UK) by dynamic light scattering method. For the morphological observation, the solution containing liposomes was dropped onto a copper mesh and stained with 2% uranyl acetate in distilled water. After 30 min of drying, the sample was observed under a TEM (Tecnai 12 Twin, FEI Company; Hillsboro, Oregon, USA) with an accelerating voltage of 120 kV. A Cryo‐electron microscope system (FEI T12 Spirit BioTwin) was also used to confirm the appearance of liposomes. Measurements were performed in triplicate in independent experiments.

The encapsulation efficiency (EE %) and drug loading of cabozantinb and IDO‐IN‐7 (DL %) were determined by ultrahigh‐performance liquid chromatography (UPLC). To determine the encapsulated cabozantinib and IDO‐IN‐7 of liposomes, methanol was added, followed by ultrasonication for 15 min for demulsification. Next, the solution was filtered through a 0.22 µm organic filter and analyzed by UPLC with a Waters ACQUITY system (Waters, Milford, MA) with a C18 column (ACQUITY, C18, 100 × 2.1 mm^2^, 1.7 µm) at room temperature at a UV absorption wavelength of 245 nm.

### Stability and In Vitro Release Behaviors

To investigate the stability under different environments, liposomes were put in PBS and serum. The stability of liposomes was assessed after storage at room temperature for 24 h. The mean vesicle size and zeta potential were measured using DLS. The dynamic dialysis method was used to assess the release behavior of cabozantinib and IDO‐IN‐7 under physiological conditions of pH in vitro. In brief, aliquots of samples were placed into a dialysis tubes (cutoff molecular weight of 3.5 kDa) and dialyzed against PBS containing 0.5% Tween‐80 (pH = 7.4). Next, the dialysis bag was stirred at 100 rpm at 37 °C, and 500 µL of dialysate was removed from the sample at predetermined intervals. To maintain a constant volume, the equal volume was replaced with fresh medium. The concentration of released cabozantinib and IDO‐IN‐7 was determined by UPLC using the Waters ACQUITY system.

### Investigation of IDO Expression Level

To evaluate the IDO expression level in the 4T1 breast tumor cell line, cells were resuspended at proper density in PBS containing Ethylenediaminetetraacetic acid (EDTA) and 0.1% bovine serum albumin (BSA). Intracellular Staining Permeabilization Wash Buffer was used to permeabilize cells following fixation with Intracellular Staining Fixation Buffer (Biolegend, San Diego, CA). After permeabilization, cells were incubated with AF647‐labeled IDO (clone 2E2/IDO, Biolegend) at 4 °C for 30 min. These samples were washed three times with cold FACS buffer. The fluorescence signal was read using flow cytometry (LSR II, BD bioscience, USA). Analysis was carried out using FlowJo 10.0 software.

### Evaluation of Cellular Uptake

As a lipophilic fluorescent probe, IR780 can be used to analyze cellular drug uptake. 4T1 cells were seeded into 24‐well plates at a density of 30 000 cells well^−1^ in complete medium containing 10% fetal bovine serum and incubated overnight. After washing twice with PBS, cells were treated with free IR780 and IR780‐loaded liposomes (IR780‐LP) at 37 °C for 4 h, where the final concentration of IR780 was 1, 4, 16 µg mL^−1^. Then, cells were washed twice with PBS and fixed with 1% w/v paraformaldehyde. For visualization, the cells were incubated with anti‐CD44‐PE for 30 min and DAPI (10 µg mL^−1^) for 5 min and washed 3 times with precold PBS. The cellular uptake images were visualized with a fluorescence microscope (DM6B, Leica, Germany). To further quantify the uptake efficiency of the cells, cells were detached by 200 µL of trypsin‐EDTA solution and centrifuged at 1000 rpm for 5 min, transferred into flow tubes (Falcon, Corning, USA) and resuspended in FACS buffer and analyzed by flow cytometry (LSR‐II, BD Biosciences, USA). Each experiment was performed in triplicate independently. The analyses were processed with FlowJo 10.0 software.

### Cytotoxicity Assay

In vitro cytotoxicity of different liposomes was measured using the MTS method according to the manufacturer's protocol (Promega, the Netherlands). 4T1 cells were seeded in 96‐well plates at 37 °C at a density of 5000 cells well^−1^ in 100 µL complete medium and incubated overnight. The empty liposome (eLP), cabozantinib solution, IDO‐IN‐7 solution, CLP, NLP, CNLP, and control group added an equal volume of blank complete medium) were then added into each well at the designated concentration of (0.3125, 0.625, 1.25, 2.5, 5, 10, 20, and 40 µm) for equivalent cabozantinib and (0.002, 0.02, 0.2, 2, 20, and 200 µg mL^−1^) eLP and incubated at 37 °C with 5% CO_2_ for 24 or 48 h. Before analysis, MTS (20 µL well^−1^) was added followed by further incubation for 1.5 h. The maximum absorbance was set at 490 nm, and the optical density of each well was scanned using a SpectraMax ABS Plus (Molecular devices, San Jose, CA). The experiments were repeated six times independently, and the half maximal inhibitory concentration (IC_50_) of the test groups was calculated using GraphPad Prism 9.0.

To explore the apoptosis‐inducing properties in vitro, 4T1 cells were seeded in 24‐well plates at a density of 50 000 cells well^−1^. After coculture with different groups of free drug or liposomes, the cells were detached by 100 µL EDTA‐free trypsin, washed twice with precooled PBS, and resuspended in binding buffer. Afterward, the cells were incubated in the dark with 5 µL Annexin V‐FITC for 15 min and 10 µL propidium iodide for 5 min. Finally, the cells were transferred to a flow tube and measured within 30 min by means of flow cytometry (LSR II, BD Biosciences, USA).

### Western Blotting Analysis

Western blotting was performed to verify the mechanism of cabozantinib. 4T1 cells were treated with 100 ng mL^−1^ HGF and cabozantinib for 24 h, washed with PBS containing a protease/phosphatase inhibitor cocktail and harvested in RIPA buffer plus inhibitor cocktail (Cell Signaling Technology). Lysates (20–40 mg) were resolved on a 4–20% SDS‐PAGE gel and transferred to a PVDF membrane (Bio‐Rad, Hercules, CA). After blocking with 5% dry milk in TBST buffer (20 mmol L^−1^ Tris‐HCl pH 7.4, 150 mmol L^−1^ NaCl, 0.1% Tween20) for 1 h, blots were probed with the following primary antibodies at 4 °C overnight: cMET (Cell Signaling Technology, 25H2), pMET (Cell Signaling Technology, Y1234/Y1235; D26), and beta‐actin (Biolegend, 2F1‐1). Blots were incubated with peroxidase‐conjugated antibody for 1 h, followed by visualization of the proteins using ECL detection reagents (Thermo Fisher, Waltham, MA).

### Cell‐Based IDO Enzymatic Activity

To evaluate the IDO1 inhibitory effect of various IDO‐IN‐7‐containing formulations, tumor cells were incubated with interferon‐gamma (IFN‐γ) (25 U mL^−1^, Thermo Fisher). Free IDO‐IN‐7, free IDO‐IN‐7 + cabozantininb, NLP, and CNLP at varying IDO‐IN‐7 concentrations were added to medium for 48 h. Afterward, the supernatant of each well (100 µL) was mixed with 30% trichloroacetic acid (50 µL) in a new 96‐well plated and kept at 50 °C for 30 min to enable complete hydrolysis of N‐formylkynurenine generated via IDO1‐mediated decomposition of essential Tryptophan. After that, supernatants of each well were mixed with an equal volume of Ehrlich reagent (2% p‐dimethylamino‐benzaldehyde w/v in glacial acetic acid) and remained at room temperature for 10 min before their absorbance at 490 nm was measured by a microreader.

### DC Maturation after Cocultured with Liposome‐Treated Cancer Cells

To assess the immunological effects of liposomes on immune cells, D1DCs were cultured in 96‐well plates with treated cancer cells for 24 h. Thereafter, supernatants were collected and subjected to IL‐12 measurement with an IL‐12p40 sandwich ELISA kit (Biolegend; San Diego, USA). D1DCs were harvested for FACS analysis. Cells were detached with PBS/EDTA (Sigma‐Aldrich, St. Louis, USA), washed with FACS buffer and stained with the following antibodies: anti‐CD11c‐APC‐eF780 (clone N418, Thermo Fisher Scientific), anti‐CD40‐APC (Clone 3/23, Biolegend), anti‐CD86‐PE‐cy7 (clone GL1, BD Biosciences), and anti‐I‐Ab (MHC class II; Clone M5/114.15.2, Thermo Fisher Scientific). DAPI was included for live‐death cell discrimination. After 30 min of staining, cells were washed with FACS buffer to remove unbound antibodies and resuspended in 100 µL FACS buffer. Flow cytometry was performed with flow cytometry (LSR‐II, BD Biosciences, USA) and data were analyzed using FlowJo 10.0 software.

### Xenograft Tumor Mouse Model of TNBC and Biodistribution Analysis

To establish the animal model, 4 × 10^5^ 4T1 cells were subcutaneously injected into the right flank of female BABL/c mice (6–8 weeks old). The tumor‐bearing mice (*n* = 4) were grouped at random in order to study the biodistribution and tumor targeting ability in vivo. Mice were intravenously administrated with free IR780 or IR780‐LP at 100 µg kg^−1^ for real‐time monitoring via the IVIS Lumina system (PerkinElmer Inc., Waltham, MA) when the tumor volume reached 200 mm^3^. Isoflurane was used for anesthesia at 1, 2, 4, 9, 12, 24, 48, and 72 h post‐treatment. Finally, the primary tumor and major organs (heart, lung, liver, spleen, and kidney) were harvested after 72 h for ex vivo imaging.

### Antitumor Efficiency Investigation

In order to evaluate the antitumor effects and safety of liposomes, mice (*n* = 8) were randomly assigned to different groups and given intravenously free drugs or liposomes every other day for a total of five injections from day 7 post‐tumor inoculation. The dose of cabozantinib and IDO‐IN‐7 were both 10 mg kg^−1^. During treatments, tumor volume and body weight were measured carefully using caliper. To calculate the tumor volume, the following formula was used: V = L × W × H (L = the length; W = the width; and H = the height of the tumor). The survival time of the mice was analyzed using Kaplan–Meier survival curves.

### Blood Analysis

To follow the immune response in the circulation, immune cells (CD3+, CD4+, CD8+, B cells) and serum IgG in the blood were investigated. 30 µL blood was collected on day 16 after the first treatments via a caudal vein puncture. For red blood cell lysis, the cells were stained with CD45.2‐APC‐eFluor780 (clone 104, Thermo Fisher), CD3‐BV421(clone 17A2, Biolegend), CD4‐BV711 (clone RM4‐5, Biolegend), CD8a‐APC‐R700 (clone 53–6.7, BD Bioscience), CD19‐BV650 (clone 6D5, Thermo Fisher), CD40‐PE (Clone 3/23, Biolegend), and CD86‐APC (clone GL1, Thermo Fisher). The cells were examined after being washed three times using flow cytometry (Aurora, Cytek, Amsterdam, the Netherlands), and the data were processed by FlowJo 10.0 software. Serum was obtained after centrifugating at 5000 rpm for 5 min, and IgG level was analyzed according to the manufacture of IgG (Total) Mouse Uncoated ELISA Kit (Invitrogen). Analysis was measured in triplicate.

### Histology and Immunohistochemistry

To further observe morphology and necrosis, the breast tumors were extracted for histological examination. Tissues were dissected, paraffin‑embedded, and sectioned (5 µm thick). Sections were stained with hematoxylin and eosin (H&E). Anti‐Ki67 mouse antibody (Invitrogen) was used for immunohistochemical (IHC) staining and performed according to the manufacturer's protocol. Briefly, paraffin‑embedded sections were deparaffinized with xylene and rehydrated in a graded ethanol series at room temperature, followed by blocked endogenous peroxidase activity for 10 min with 0.3% H_2_O_2_ in dH_2_O. After being washed with dH_2_O, sections were pretreated with boiling citrate buffer for 10 min. Then sections were blocked in 5% bovine serum albumin in PBS for 30 min and then incubated with primary antibodies (anti‐Ki67) at 4 °C overnight. Following three washes in 0.1% Tween/PBS, sections were incubated with the secondary antibody Impress goat antirat IgG (HRP) at room temperature for 30 min and incubated with DAB system for 1 min. The immunoreaction was quenched when brown precipitates formed following incubation in diaminobenzidine. Sections were subsequently counterstained with Mayer's hemalum solution 1:4 in dH2O for 1 min. Finally, sections were viewed by Pannoramic 250 (3DHISTECH, Budapest, Hungary).

### Tumor Microenvironment Analysis

The tumor, spleen, tumor‐draining lymph nodes, and nontumor‐draining lymph nodes were examined by sacrificing mice and collecting these tissues on day 16. The resected tumors were cut into small pieces with sterile scissors and forceps, and them incubated with Liberase TL (Roche, Mannheim, Germany) in serum‐free RPMI 1640 medium at 37 °C for 30 min. Single‐cell suspensions were acquired from tissues by gently grinding the fragments through a 70 µm cell strainer (Falcon, NY). Prior to staining, the spleen cell suspension was washed with lysis buffer twice to remove red blood cells. All the cells were then equally divided and stained with two distinct antibody panels. The lymphoid maker panel included: anti‐CD45.2‐PerCP‐Cy5.5 (clone 104, Thermo Fisher), anti‐CD3e‐BV421 (clone 17A2, Biolegend), anti‐CD4‐BV605 (clone RM4‐5, Biolegend), anti‐CD8α‐APC‐R700 (clone 53–6.7, BD Bioscience), anti‐CD19‐BV650 (clone 6D5, Thermo Fisher), anti‐GranB‐APC (clone QA16A02, Biolegend), anti‐IFN‐γ‐PE‐Cy7 (clone B27, Biolegend), and anti‐TNF‐α‐FITC (clone MP6‐XT22, Biolegend). The myeloid marker panel included: anti‐CD11b‐eFluo450 (clone M1/70, BD Biosciences), anti‐F4/80‐PE‐Cy5 (clone BM8, Biolegend), anti‐Ly6G‐BV785 (clone 1A8, Biolegend), anti‐Ly6C‐BV605 (clone HK1.4, Biolegend), anti‐CD11c‐APC‐eFluo780 (clone HL3, Thermo Fisher), anti‐CD40‐PE (clone 3/23, BD Biosciences), anti‐CD86‐APC (clone GL1, Thermo Fisher), anti‐iNOS‐AF488 (clone CXNFT, Thermo Fisher), and anti‐CD206‐AF647 (clone C068C2, Biolegend). The expression of the cell markers was analyzed by flow cytometry (Aurora, Cytek, Amsterdam, the Netherlands) and the data were analyzed with FlowJo 10.0 software.

### ELISA

Serum was obtained after centrifuge at 5000 rpm for 5 min, and cytokine levels of IFN‐γ, tumor necrosis factor alpha (TNF‐α), interleukin (IL)−6, IL‐10, IL‐12were analyzed using an ELISA kit (Biolegend) according to the manufacture. Analysis was measured in triplicate.

### Statistical Analysis

Data were presented as mean ± standard deviation. One‐ and two‐way analysis of variance was used when making multiple comparisons. Bonferroni posttests were performed when comparing all groups. A two‐tailed *t*‐test was used when comparing two groups. In vivo tumor treatment studies were repeated in two independent experiments to ensure adequate sample size and reproducibility. All statistical analysis was performed using GraphPad Prism software. Statistical significance was noted as follows: *, *p* < 0.05, **, *p* < 0.01, ***, *p* < 0.001, ****, *p* < 0.0001.

## Conflict of interest

The authors declare no conflicts of interest.

## Supporting information

Supporting Information

## Data Availability

Data sharing is not applicable to this article as no new data were created or analyzed in this study.
