# Supplementary material for: Dual‐Targeting Nanoliposome Improves Proinflammatory Immunomodulation of the Tumor Microenvironment
Source: Adv Healthc Mater. 2023 Sep 21;12(31):2302046. doi: 10.1002/adhm.202302046 (PMC11468610; doi:10.1002/adhm.202302046)
Supplement: Supplementary file 1 — Supporting Information [file ADHM-12-2302046-s001.pdf]

ADVANCED
HEALTHCARE
MATERIALS

Supporting Information

for *Adv. Healthcare Mater.*, DOI 10.1002/adhm.202302046

Dual-Targeting Nanoliposome Improves Proinflammatory Immunomodulation of the Tumor Microenvironment

Zili Gu, Candido G. da Silva, Sen Ma, Qi Liu, Timo Schomann, Ferry Ossendorp and Luis J. Cruz**

Dual-Targeting Nanoliposome Improves Pro-inflammatory Immunomodulation of the Tumor Microenvironment

Zili Gu[1], Candido G. Da Silva[1], Sen Ma[2], Qi Liu[3], Timo Schomann[1,4], Ferry Ossendorp[5*], Luis J. Cruz[1,*]

[1]Department of Radiology, Leiden University Medical Center, the Netherlands

[2]Department of Ophthalmology, Leiden University Medical Center, the Netherlands

[3]Department of Internal Medicine, University of Texas Southwestern Medical Center, Dallas, Texas, USA

[4]Department of Vascular Surgery, Leiden University Medical Center, the Netherlands

[5]Department of Immunology, Leiden University Medical Center, the Netherlands

[*] **Corresponding author:** l.j.cruz_ricondo@lumc.nl (L.J. Cruz); F.A.Ossendorp@lumc.nl (F.A.Ossendorp)

Table S1 Physicochemical properties of liposomes

Liposomes	Diameter (nm)	PDI	Zeta potential
**eLP**	124.43 ± 2.27	0.068 ± 0.019	-7.11 ± 5.69
**CLP**	122.97 ± 1.56	0.067 ± 0.025	-4.42 ± 8.88
**NLP**	124.07 ± 0.69	0.054 ± 0.019	-5.67 ± 8.98
**CNLP**	115.53 ± 2.35	0.140± 0.013	-7.61 ± 6.37

Table S2 Encapsulation efficiency (EE) and drug loading (DL) of liposomes

Liposomes	Cabozantinb		NLG919	
	EE(%)	DL(%)	EE(%)	DL(%)
**CLP**	80.58 ± 1.04	3.77 ± 0.05	--	--
**NLP**	--	--	91.64 ± 2.04	4.31± 0.10
**CNLP**	78.21 ± 1.27	3.66 ± 0.06	89.55 ± 0.79	4.21 ± 0.44

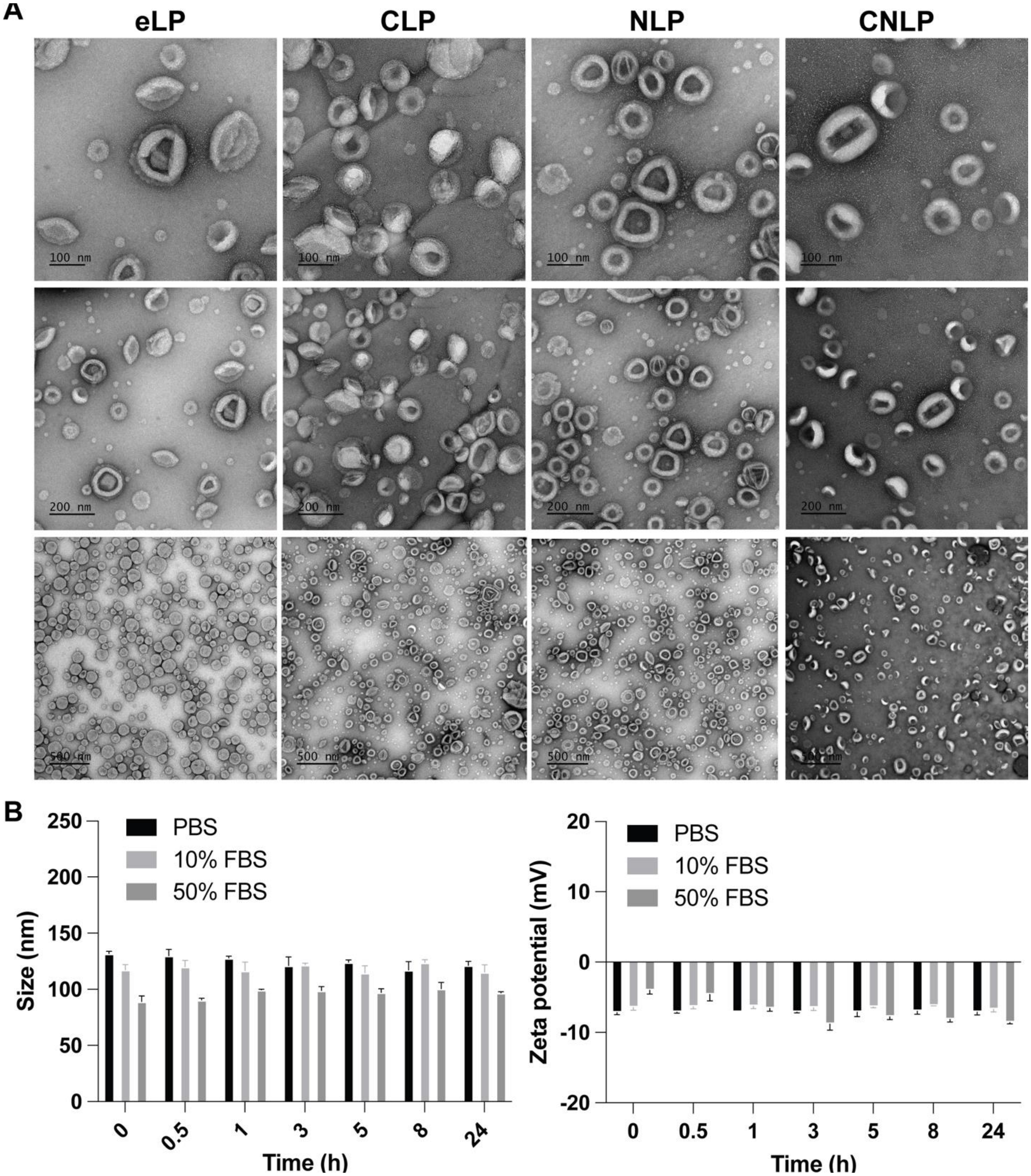

Fig. S1. A. TEM Photos of eLP, CLP, NLP, and CNLP. B. The Stability of liposomes in PBS and serum (n=3, results were shown in mean ± S.D., *, $p<0.5$, **, $p<0.1$, ***, $p<0.01$, ****, $p < 0.0001$).

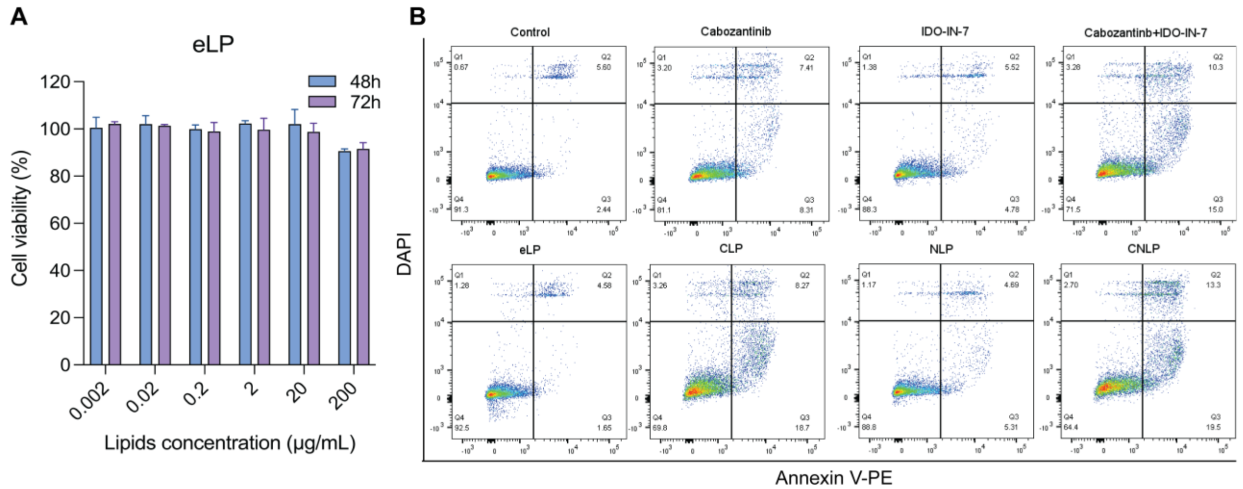

Fig. S2. Cytotoxicity of empty liposomes (eLP) and quantification of apoptosis investigation in 4T1 cells (n=3, results were shown in mean ± S.D., *, p<0.5, **, p<0.1, ***, p<0.01, ****, p < 0.0001).

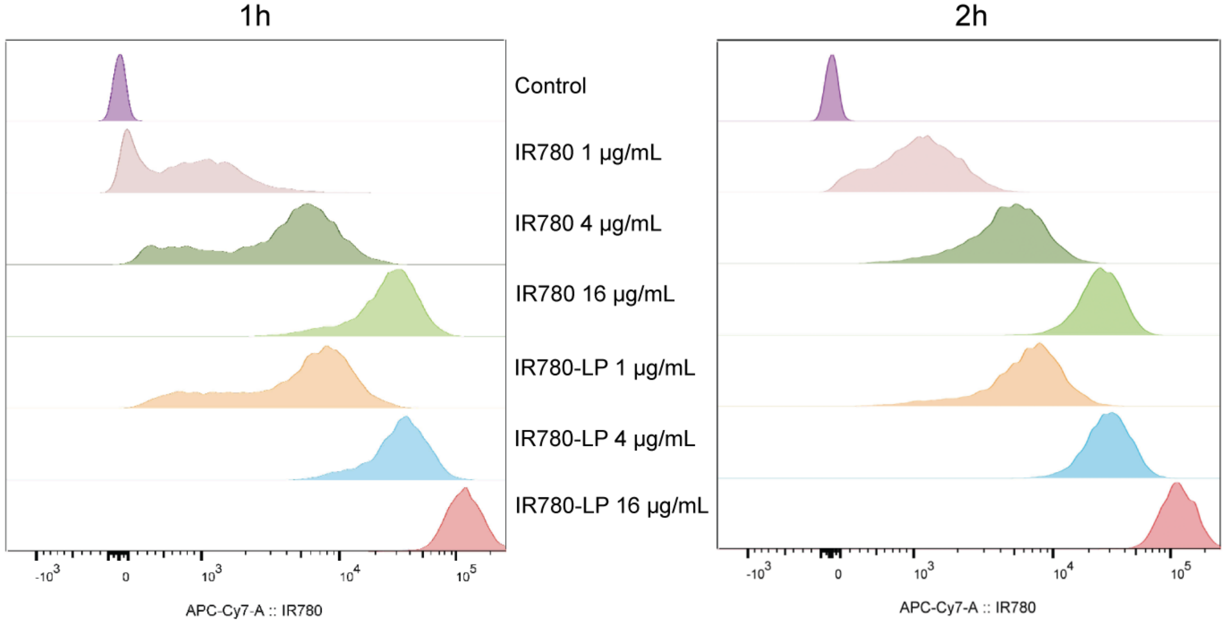

Fig. S3. Histogram of intracellular uptake after incubated with free IR780 and IR780-LP after 1 & 2 h in 4T1 cells *in vitro*.

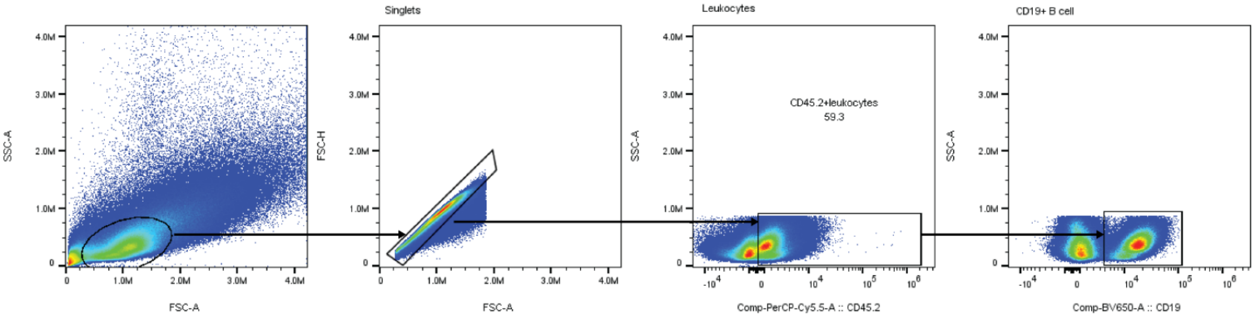

Fig. S4. Gating strategy for B cells.

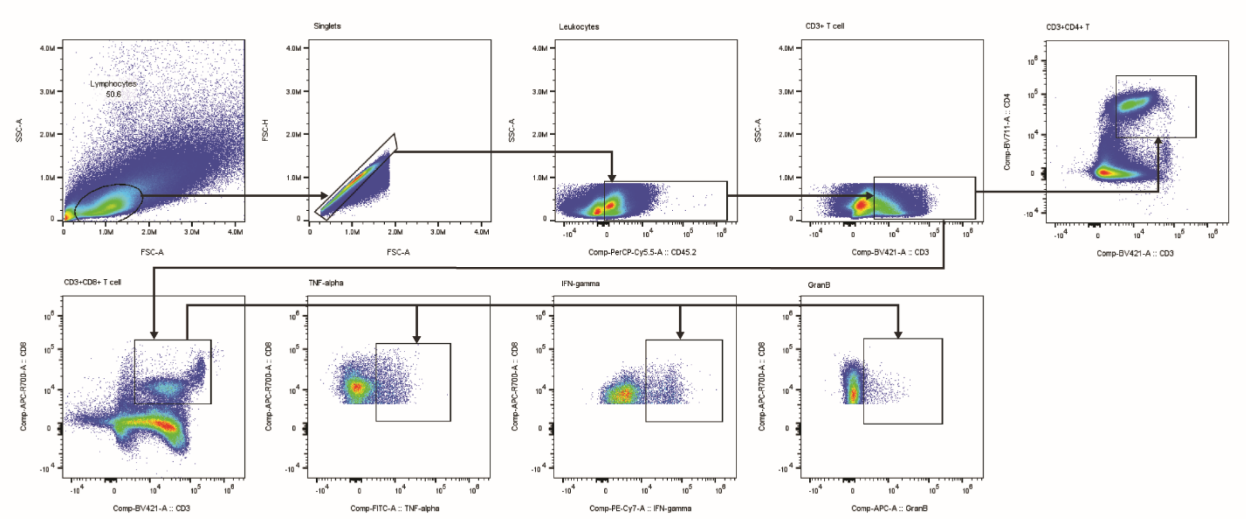

Fig. S5. Gating strategy for lymphoid populations.

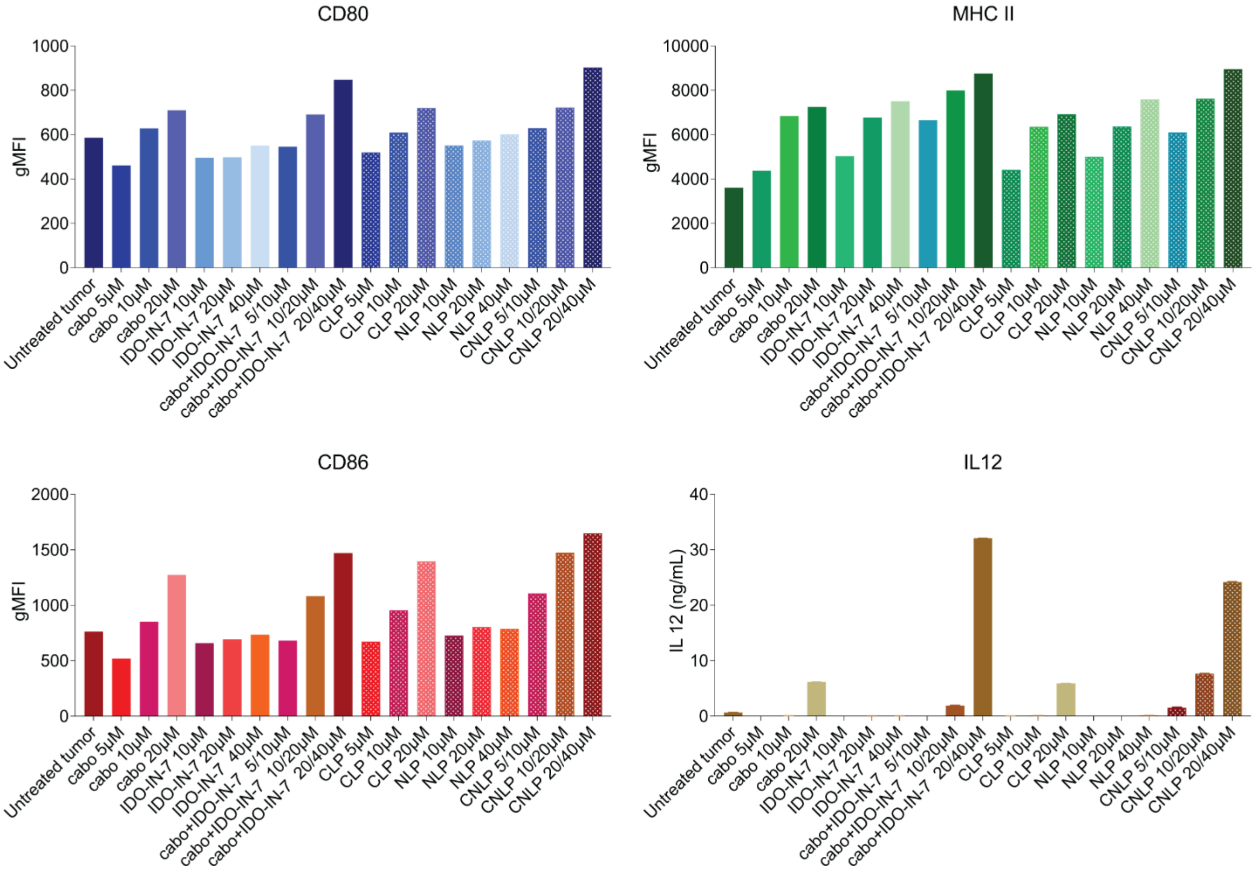

Fig. S6. DC maturation after co-cultured with drug- or liposome-treated tumor cells.

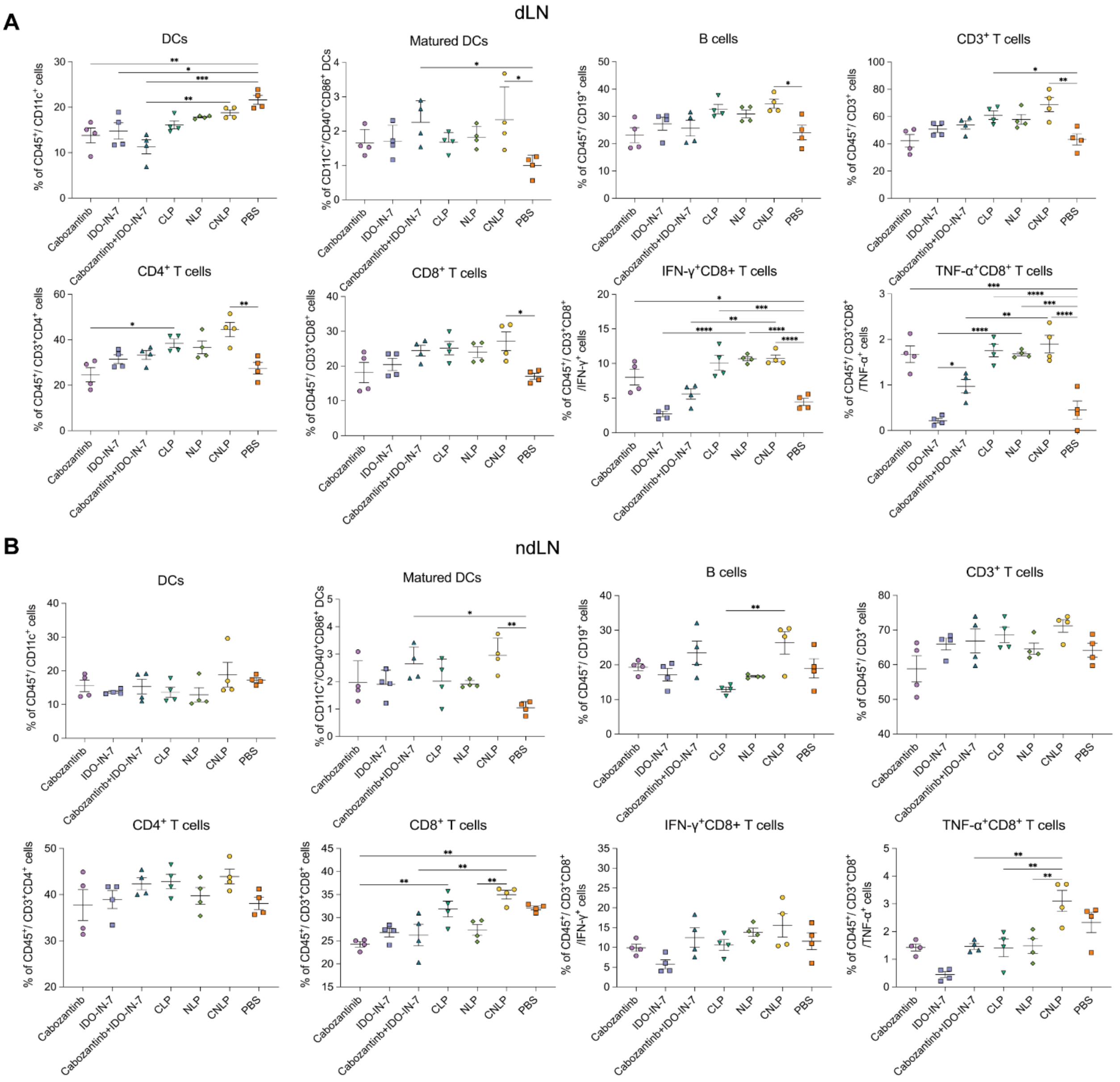

Fig. S7. **The level of lymphatic populations in the lymph nodes after treatments.** A. Percentage of DCs, matured DCs, B cells, $CD3^+$, $CD4^+$, $CD8^+$, IFN-$\gamma^+$$CD8^+$, TNF-$\alpha^+$$CD8^+$ T cells in the dLN. B. Percentage of DCs, matured DCs, B cells, $CD3^+$, $CD4^+$, $CD8^+$, IFN-$\gamma^+$$CD8^+$, TNF-$\alpha^+$$CD8^+$ T cells in the ndLN.

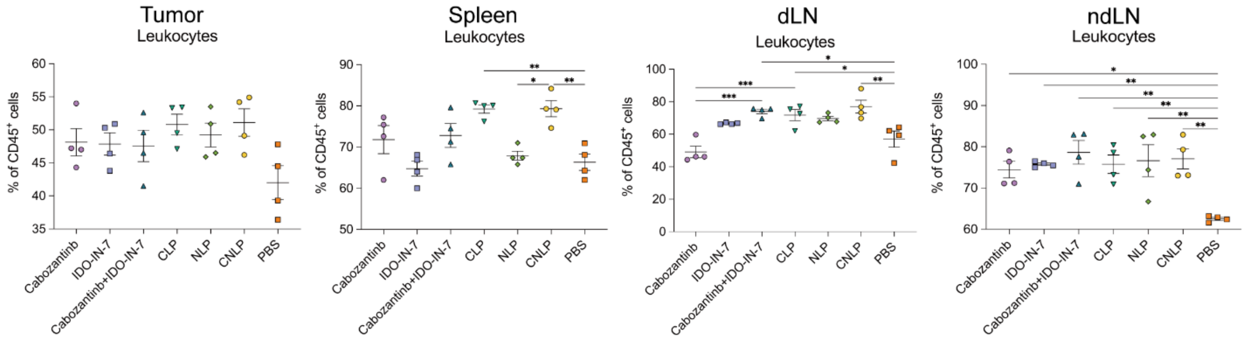

Fig. S8. The level of CD45+ leukocytes in tumor microenvironment, spleen, dLN, and ndLN (n=4, results were shown in mean ± S.D., *, p<0.5, **, p<0.1, ***, p<0.01, ****, p < 0.0001).

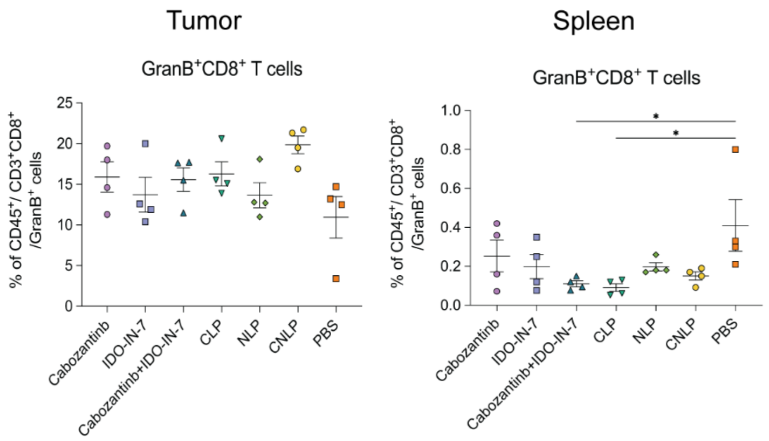

Fig. S9. The level of Granzyme B^{+} $CD8^{+}$ T cells in tumor microenvironment and spleen (n=4, results were shown in mean ± S.D., *, p<0.5, **, p<0.1, ***, p<0.01, ****, p < 0.0001).

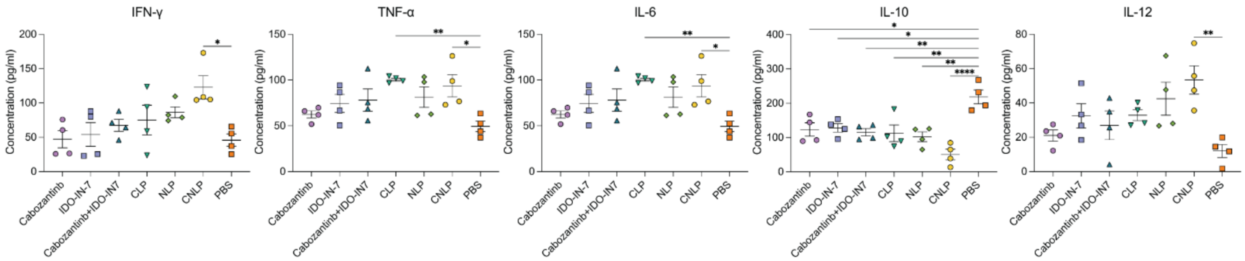

Fig. S10. The level of cytokines in the tumor after the administration of different treatment (n=4, results were shown in mean ± S.D., *, p<0.5, **, p<0.1, ***, p<0.01, ****, p < 0.0001).

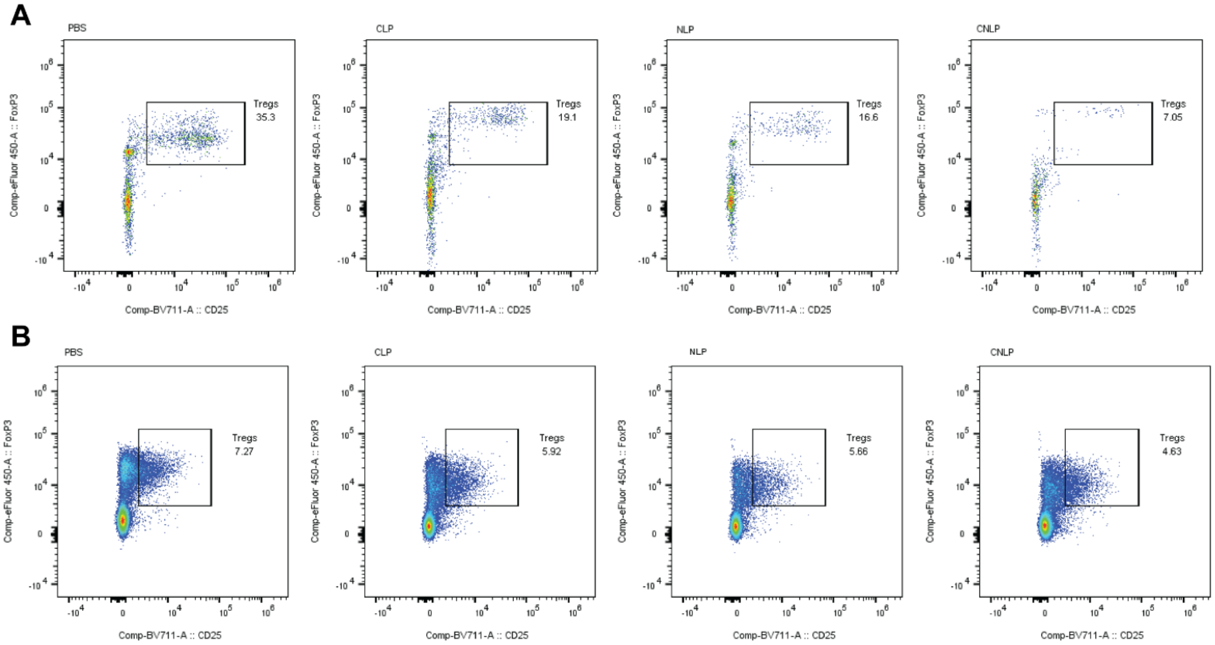

Fig. S11. T_{reg} population in the tumor microenvironment and spleen receiving liposomal treatment.

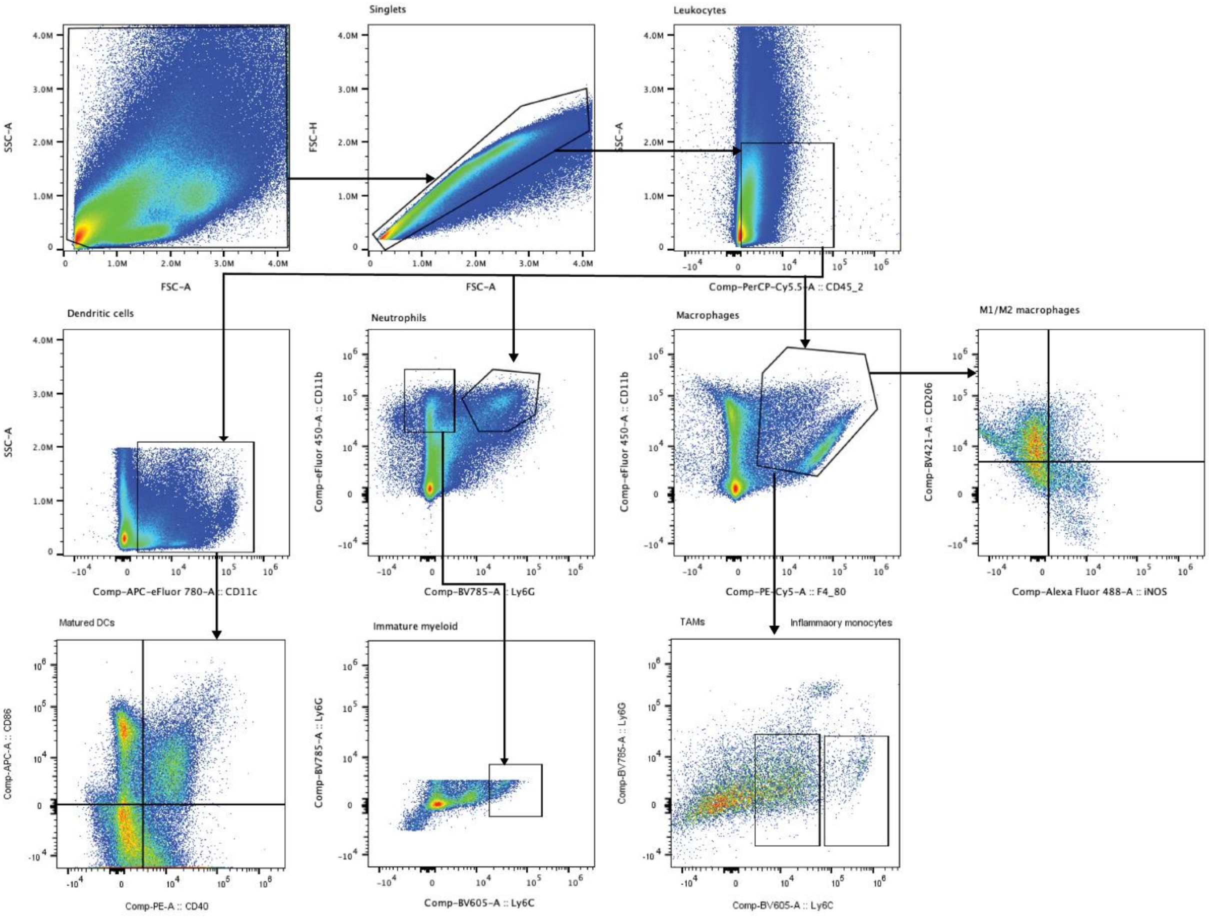

Fig. S12. Gating strategy for myeloid populations.

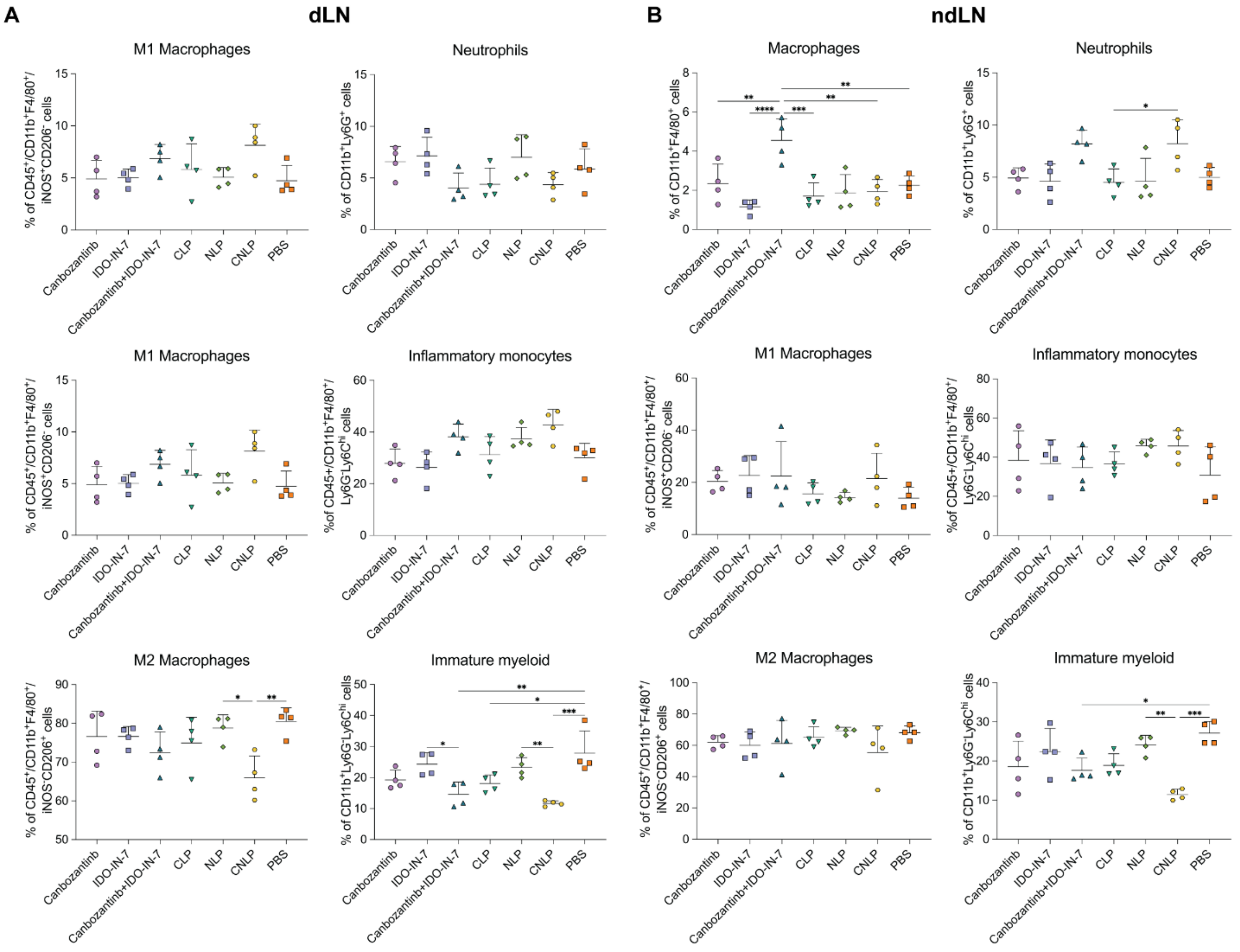

Fig. S13. **The level of myeloid populations in the lymph nodes treatments.** A. Percentage of macrophages, M1&M2 macrophages, neutrophils, inflammatory monocytes, and immature myeloid in the dLN. B. Percentage of macrophages, M1&M2 macrophages, neutrophils, inflammatory monocytes, and immature myeloid in the ndLN.
